# Phosphate Recovery from Urine-Equivalent Solutions for Fertilizer Production for Plant Growth

**DOI:** 10.1021/acssuschemeng.3c03146

**Published:** 2023-11-01

**Authors:** Marina Avena Maia, Olaf Prosper Kranse, Sebastian Eves-van den Akker, Laura Torrente-Murciano

**Affiliations:** † Department of Chemical Engineering and Biotechnology, University of Cambridge, Philippa Fawcett Drive, CB3 0AS Cambridge, U.K.; ‡ Crop Science Centre, Department of Plant Sciences, University of Cambridge, CB3 0LE Cambridge, U.K.

**Keywords:** adsorption, phosphate recovery, wastewater treatment, phosphate adsorption mechanism

## Abstract

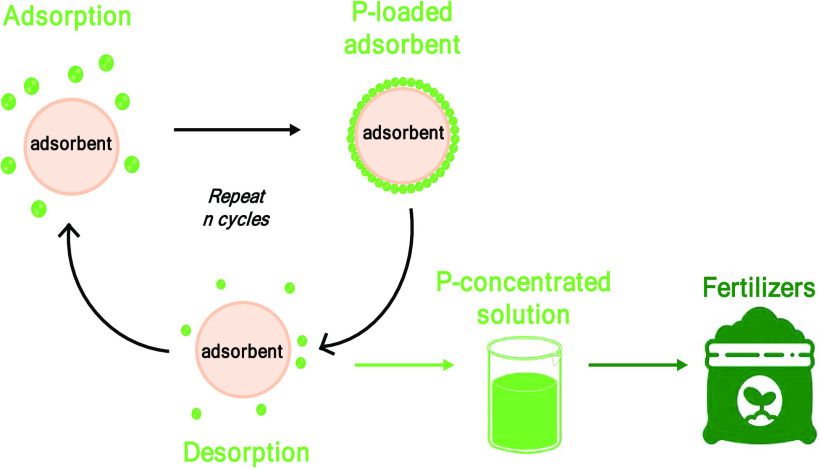

This study presents a proof of concept for the recovery of phosphate from aqueous solutions with high phosphorus (PO_4_–P) initial contents to simulate the concentration of streams from decentralized wastewater systems. Solutions with ∼500 ppm phosphorus enable phosphate adsorption and recovery, in contrast to the highly diluted inlet streams (<10 ppm) from centralized wastewater treatment plants. In this work, Mg–Fe layered double hydroxide is used as a phosphate adsorbent, demonstrating its separation from aqueous streams, recovery, and use as a fertilizer following the principles of circular economy. We demonstrate that the mechanism of phosphate adsorption in this material is by a combination of surface complexation and electrostatic attraction. After the loss of crystallinity in the presence of water in the first cycle and its associated decrease in adsorption capacity, the Mg–Fe layered double hydroxide (LDH) is stable after consecutive adsorption/desorption cycles, where desorption solutions were reused to substantially increase the final phosphate concentration demonstrating the recyclability of the material in a semicontinuous process. Phosphate recovered in this way was used to complement phosphate-deficient plant growth medium, demonstrating its efficacy as a fertilizer and thereby promoting a circular and sustainable economy.

## Abstract 2

Proof of concept for the recovery of phosphate from concentrated aqueous solutions with its recycling and reuse as a fertilizer product.

## Introduction

Phosphorus is one of the three macronutrients needed by plants for development and growth. Together with nitrogen (N) and potassium (K) nutrients, phosphorus (P) plays a leading role in NPK fertilizers that are essential to maximize crop productivity.^1^ Fertilizer production is by large the main user of phosphorus globally, accounting for between 80 and 90% of the total world demand.^2^ According to the Food and Agriculture Organization of the United Nations (FAO), in 2021, the phosphorus fertilizer demand was 48.3 Mtn.^3^ It is expected to reach up to 12.4 kgP/ha·yr by 2050, an increase of approximately 50% with respect to 2005.^4^ As a result, the fertilizer industry has reported investments of over US$40 billion due to this expected increase.^5^ Phosphorus is a finite resource originated from igneous rocks and marine sedimentary deposits, obtained at industrial scale by mining. Phosphate rocks reserves are present in a limited number of countries, with large parts of the world, including Europe, being almost completely dependent on imports. The largest sedimentary deposits of phosphate rocks are found in northern Africa, China, and the United States.^6,7^ Morocco alone controls the majority of the global supply, holding approximately 70% of the world’s phosphate rock reserves, followed by China, which holds only 4.5% of the reserves.^8^ In 2021, 220 Mtn of phosphate rocks were mined from the finite reserves worldwide. Considering that 30% of the weight of phosphate rock is P_2_O_5_, it can be estimated that approximately 28 Mtn of phosphorus was extracted.^8^ The lack of geographical distribution of these reserves creates a challenging scenario to sustain a reliable phosphate rock supply, subject to geopolitical and economical disruptions in addition to depletion of the reserves. In fact, due to their high-economic importance and nonsubstitutable nature, phosphate rocks have been declared as one of the 30 critical resources in the European Union.^9^

Phosphorus plays an important role in mammals, not only for bone health but also for the growth and maintenance of cells and tissues, being present in the adenosine triphosphate (ATP) molecule.^10^ The excess of phosphorus is released in urine. In particular, human urine from households contributes largely to the amount of nutrients found in wastewater streams. Approximately, ∼50% of the phosphate mass load in municipal wastewater treatment plants comes from human urine.^2,11^ The remainder of phosphate in streams is originated from residual waste generated by animal feed supplements and fertilizer industries, food and drinks applications, and detergent production.^2^ Human urine consists primarily of water and contains many compounds in varying concentrations, which are dependent on the diet and individual lifestyles. The main organic components include urea (9300–23,300 mg/L), creatinine (670–2150 mg/L), hippuric acid (50–1670 mg/L), uric acid (40–670 mg/L), and inorganic ions such as chloride (1870–8400 mg/L), sodium (1170–4390 mg/L), potassium (750–2610 mg/L), phosphorus (250–1070 mg/L), and sulfates (163–1800 mg/L).^12,13^ One of the main challenges for the recovery of phosphorus from urine is the fact that it is heavily diluted with other waste streams down to 5 and 20 mg/L of phosphorus (∼90% dilution)^14,15^ by the time it reaches centralized waste water treatment plants. In addition, in such plants, the focus is phosphate removal and not its recovery, with an energy requirement for phosphate extraction through chemical precipitation approximately 49 MJ kg^–1^ P (i.e.,13.6 kWh/kg of P).

A completely new alternative approach for its recovery is the deployment of decentralized wastewater systems (i.e., no-mix toilets) to collect and treat urine as a separate waste stream, offering the opportunity to recover phosphate more efficiently at the production point, avoiding its dilution.^16^ The applicability of urine diversion toilets as an alternative to centralized systems started diffusing from 2010 onward mostly in developing countries, where access to proper sanitation is needed. A large-scale rural and peri-urban sanitation program in Durban, South Africa, was implemented by Bill and Melinda Gates Foundation.^17^ In this program, 75,000 urine diversion toilets serving 450,000 inhabitants were installed. In addition, the International Federation of Red Cross and Red Crescent Societies (IFRC) implemented through the WASH program (Water, Sanitation and Hygiene) the installation of urine diversion toilets in different countries: ∼900 in Bolivia and ∼1000 each in Kenya, Burkina Faso, and Uganda.^18^ Urine diversion toilets have also been employed in areas with sewer systems. Separett, a Sweden-based company, has sold over 100,000 urine diversion toilets across the world.^19^ Through the application of urine diversion toilets, a few studies evaluated the potential of nutrient extraction directly from human urine,^20−22^ where phosphate was recovered as a possible fertilizer product. It is important to note that when phosphate-based fertilizers are precipitated directly from human urine, other components can contaminate the recovered final product, affecting its application in agriculture. Urine has a complex matrix with other species, including micropollutants,^20^ such as caffeine, anti-inflammatory drugs and antibiotics, heavy metals, and high salt concentration.^23^ These compounds are harmful to be applied directly in soils since they reduce crop growth and can stop plant reproduction.^24,25^ Alternatively, phosphate can be first separated from urine and subsequently recovered as a fertilizer to avoid contamination with other compounds. This new perspective re-evaluates human waste as a reusable and eco-friendly resource in alignment to the 2020 European Commission’s Circular Economy Action Plan to reduce waste and ensure a more sustainable application and reuse of raw materials.^26^ Phosphorus recovery from urine and its subsequent application as a fertilizer promote a circular and sustainable closed loop of nutrients for their reutilization in agriculture. At the same time, phosphate recovery initiatives can decrease P content in water bodies, reducing environmental hazards caused by eutrophication.

Current phosphate recovery approaches consist of its extraction from the liquid phase or from the sludge waste generated at centralized wastewater treatment plants.^14,27−28,29^ The latter recovers the phosphate fixed in the sewage sludge phase and/or sewage sludge ash through wet-chemical and thermochemical treatments. In the wet-chemical process, the bound phosphate in the sludge is released back into the liquid phase by the addition of strong acids or alkalis. In the thermochemical process, chloride additives such as NaCl, KCl, MgCl_2_, and CaCl_2_ are mixed with the sludge at high temperatures (800–1000 °C) to produce volatile heavy metal chlorides and the extraction of phosphorus species.^28^ After recondensation with no heavy metal contamination, chemical precipitation or adsorption processes are used to recover phosphates from the supernatant. In the precipitation process, the dissolved phosphate is crystallized in the form of different salts with a low water solubility. Common products obtained from phosphate precipitation are hydroxyapatite (Ca_5_(OH)(PO_4_)_3_), struvite (MgNH_4_PO_4_·6H_2_O), K-struvite (KMg(PO_4_)·6(H_2_O)), and vivianite (Fe_3_(PO_4_)_2_·8H_2_O).^29^ Particularly for fertilizer applications, struvite is an interesting recovery product since it can be directly used as a slow-release fertilizer.^30−32^ The main limitation of the use of chemical precipitation directly in inlet wastewater streams is its initial low concentration in phosphorus. An alternative is enrichment by using selective adsorption. An extensive number of adsorbents have been developed and/or applied for phosphate separation including metal hydroxides and oxides, clays, zeolites, mesoporous silica-based materials, and activated carbon.^33−38^ Among them, metal oxides and hydroxides, in particular metallic layered double hydroxides, have shown high phosphate adsorption capacity due to their positive ζ-potential values in a wide range of pH, which facilitates the electrostatic attraction between the adsorbents and negatively charged phosphate species.^39,40^ Despite the potential of this approach, previous studies focused almost exclusively on phosphate adsorption in highly diluted streams (<10 mg/L)^41−44^ representative of the concentrations of inlet streams in centralized wastewater treatment plants. As a consequence, these studies evaluate the adsorption–desorption process at different points of the isotherm. Although most of previous studies on the adsorption of phosphate on layered double hydroxide (LDH) materials have been performed using model solution containing solely phosphate, scattered studies suggest the detrimental effect of the presence of some anions such as sulfate and chloride^41,43^ and citrate^45^ on the adsorption capacity.

In this work, we present the feasibility of a phosphate recovery process for decentralized systems (i.e., production points) aligned to the principles of circular economy. Consecutive adsorption/desorption cycles using a Mg–Fe layered double hydroxide enrich model aqueous phosphate solutions with an initial concentration of 500 mg/L phosphorus to simulate human urine typical values by reusing the desorption solution. Phosphorus is then recovered and assessed for its use as a fertilizer using the model species *Arabidopsis thaliana* through a novel method. This work challenges the current inefficient phosphate recovery processes, providing a novel approach for the recovery of this limited resource in a process less energy-intensive than that currently used in wastewater treatment plants.

## Experimental Section

### Synthesis and Characterization of Mg–Fe Layered Double Hydroxide

MgFe–CO_3_ layered double hydroxide (LDH) with a targeted Mg/Fe ratio of 3:1 was synthesized using a coprecipitation method at a variable pH.^46^ The 3:1 Mg/Fe ratio was chosen to promote the layered hydrotalcite-like structure based on previous studies.^43,47,48^ The coprecipitation was conducted in a glass stirred vessel at room temperature initially containing 50 mL of aqueous 2 M Na_2_CO_3_. A solution of the metallic salts (Mg(NO_3_)_2_·6H_2_O and Fe(NO_3_)_3_·9H_2_O) with a total metal concentration of 1 M and the targeted Mg/Fe ratio was added to the Na_2_CO_3_ solution through a peristaltic pump at a flow rate of 5 mL/min. The pH of the solution was maintained at a range between 9.5 and 12 to guarantee the precipitation of the two metallic species and the formation of the layered structure. After 10 min, the resulting slurry was stirred for crystallization at room temperature for 24 h. The obtained precipitate was collected by centrifugation and washed four times with 400 mL of Milli-Q water. The wet solid was dried at 80 °C under vacuum overnight and milled down to 180 μm by using a sieve.

Surface area and porosity measurements were determined by N_2_ physisorption at 77 K using the Micromeritics ASAP 2020 equipment. The samples were pretreated at 120 °C for 6 h under ultrahigh vacuum before the analysis. The specific surface area (*S*_BET_) was calculated according to the standard Brunauer–Emmett–Teller (BET) method, and the pore size distribution was determined by the Barrett–Joyner–Halenda (BJH) method using the desorption data.

Attenuated total reflection Fourier transform IR (ATR-FTIR) of the samples, before and after adsorption, were obtained using a PerkinElmer FTIR/NIR Frontier spectrophotometer in the wavelength range 4000–600 cm^–1^ at room temperature, with 4 cm^–1^ resolution and 32 scans.

Powder X-ray diffraction (XRD) patterns were collected using a Bruker D8-QUEST PHOTON-100 diffractometer, between 2 and 80° 2θ using Cu K_α_ radiation (λ = 1.5418 Å) with increments of 0.02° and 80 s per step.

The ζ-potential measurements of Mg–Fe LDH in suspension before and after adsorption at a pH range between 3 and 12.5 were measured by using a ζ-potentiometer Zetasizer Nano-ZS Malvern system. The pH of this dispersion was adjusted by 0.1 M NaOH and 0.1 M HCl.

To evaluate the possible metal (Mg and Fe) dissolution of the LDH material during hydrolysis and/or adsorption, the solid was filtered and the supernatant solution was analyzed using a Thermo Fisher Scientific iCAP7400 Duo ICP-OES spectrometer with argon as the torch gas and nitrogen for purging.

### Adsorption/Desorption Experiments

Batch adsorption tests were carried out at 25 °C by using 10 mL of an aqueous solution with an initial phosphorus concentration of 500 mg/L to simulate the concentration in urine. The model solutions were prepared by dissolving KH_2_PO_4_ in Milli-Q water. Unless otherwise stated, the concentration of Mg–Fe LDH was 5 g/L, which was chosen based on preliminary experiments that evaluated the optimal adsorbent concentration. The effect of pH on phosphate adsorption was evaluated by performing experiments in a pH range between 4 and 10. The pH of the solutions was adjusted by 0.1 M NaOH and 0.1 M HCl. The effect of the ionic strength on phosphate adsorption was analyzed by using NaCl as an electrolyte at different concentrations of 0.01 and 0.1 M. To evaluate the effect of phosphate initial concentration, adsorption experiments with different concentrations of phosphate ranging from 40 to 1500 mg/L were performed. The effect of other species commonly present in urine on the phosphate adsorption capacity was evaluated by adding them individually to a model 900 mg/L phosphate aqueous solution. The concentration of each studied species is as follows, taking into consideration their average concentration in urine: KCl = 1600 mg/L, urea = 25,000 mg/L, Na_2_SO_4_ = 2300 mg/L, and NaCl = 4600 mg/L.

The concentration of phosphate in the solution during the adsorption tests was determined according to an APHA Standard Colorimetric Method 4500-P C (vanadomolybdophosphoric acid colorimetric method).^49^ The vanadate-molybdate reagent was made by dissolving 0.625 g of ammonium metavanadate through heating to boiling in 150 mL of Milli-Q water. After the solution was cooled, 165 mL of concentrated 37% HCl was added. Then, the solution was cooled again to room temperature, and 12.5 g of ammonium molybdate was added into the solution. The final solution was diluted to 500 mL. In a typical analysis, 0.5 mL of the vanadate-molybdate reagent was added to 2.5 mL of the phosphate-containing solution, forming a vanadomolybdophosphoric acid with a characteristic yellow color. The absorbance of the solution at a wavelength of 421 nm was measured by using a Cary 60 UV–vis-NIR spectrophotometer. The phosphorus concentration (PO_4_–P) was calculated using a calibration curve of standard solutions, with concentrations between 1 and 50 mg/L (Figure S1). When needed, the samples were diluted to fit into the concentration range of the calibration curve. All of the experiments were carried out in triplicate, and the associated experimental error was calculated through the standard deviation obtained from the three experiments. The phosphate adsorption capacity (*q*_e_) and removal efficiency (*R* %) were determined by eqs 1 and 2.
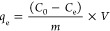
1
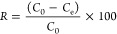
2where *C*_0_ and *C*_e_ are the initial and equilibrium concentration of phosphate (PO_4_–P) in solution (mg/L), *m* is the adsorbent dry weight (g), and *V* is the suspension volume (L).

**Figure 1:**
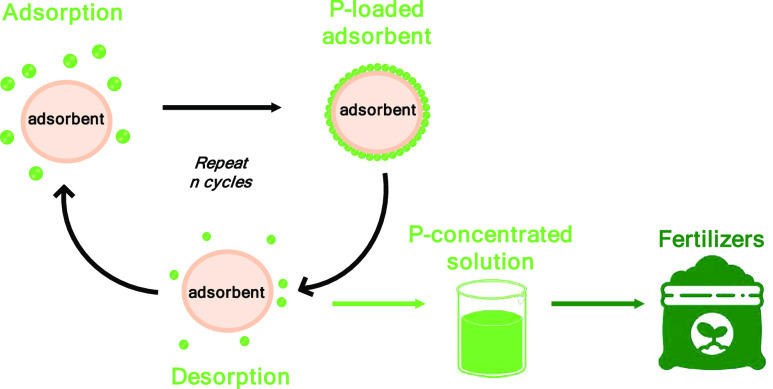
Schematic representation of the adsorption–desorption process for phosphate recovery.

After adsorption, the phosphate-loaded adsorbent was separated through centrifugation from the solution and when indicated reused for the desorption and recyclability studies. For the desorption step, the adsorbent was suspended into a 10 mL NaOH 0.5 M solution with a pH value of 13.3 at 25 °C for 30 min and the phosphorus concentration in the solution was measured as indicated above. The recovery adsorbent yield, defined as the amount of solid adsorbent recovered after each cycle, is maintained between 98 and 100% after each cycle. The same desorption solution was reused in each desorption cycle as depicted in Figure 1. The reuse of the desorption solution in multiple desorption cycles enriches the phosphate concentration, minimizing the volume of solutions processed for struvite crystallization. It also opens the door for a recycle of the solution, minimizing the production of waste.

The desorption efficiency (*D*_e_) is defined as the percentage of phosphate desorbed according to eq 3.
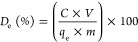
3where *C* is the concentration of phosphate (PO_4_–P) in the desorption solution (mg/L), *V* is the volume of the NaOH desorption solution (L), *q*_e_ is the initial amount of phosphate adsorbed onto the adsorbent (mg/g), and *m* is the amount of adsorbent used in the desorption experiments (g).

The phosphate-rich reclaimed solution from 5 adsorption–desorption cycles was precipitated to form struvite (MgNH_4_PO_4_·6H_2_O). For this, magnesium and ammonium chloride salts (MgCl_2_ and NH_4_Cl) were added to the phosphate solution at a molar ratio of Mg/NH_4_/PO_4_ = 1:1:1 and at a pH of 8.5 adjusted using a 0.1 M HCl solution. Scanning electron microscopy (SEM) of the synthesized struvite was carried out using a TESCAN MIRA 3 at a 5 kV accelerating voltage and a working distance of 7.8 mm. Samples were sputter-coated with approximately 10 nm layer of platinum to minimize charging.

### Plant Growth and Leaf Surface Area Calculation

To assess the viability of the synthesized struvite as a fertilizer, three different growth media were prepared: (1) 1/2 Murashige & Skoog (1/2 MS, Duchefa); (2) Mg- and P-deficient medium (MgP-DM), a media based on 1/2 MS media, lacking key plant growth macronutrients (Table 1); and (3) MgP-DM with 0.1534 g/L of the recovered struvite (MgP-DMS). The Mg- and P-deficient medium was calculated so that the addition of the aforementioned struvite balances, as much as possible, the ion composition to 1/2 MS. Importantly, SO_4_^2–^ and H^+^ were not considered for ion balancing in the MgP-DMS media, and there was 0.2 mM less Mg^2+^ compared to 1/2 MS. The difference between 1/2 MS and MgP-DM is the complete omission of magnesium and phosphorus. All three media are shown in Table 1.

**Table 1:** Different Media Compositions Tested in the Plant Growth of*Arabidopsis thaliana*

media name	1/2 MS	Mg- and P-deficient medium (MgP-DM)	Mg- and P-deficient medium (MgP-DMS)
chemical formula	concentration (mg/L)	concentration (mg/L)	concentration (mg/L)
CoCl_2_·6H_2_O	0.013	0.013	0.013
CuSO_4_·5H_2_O	0.012	0.012	0.012
FeNaEDTA	18.35	18.35	18.35
H_3_BO_3_	3.1	3.1	3.1
KI	0.42	0.42	0.42
MnSO_4_·H_2_O	8.45	8.45	8.45
Na_2_MoO_4_·2H_2_O	0.13	0.13	0.13
ZnSO_4_	2.42	2.42	2.42
CaCl_2_·2H_2_O	219.8	219.8	219.8
KH_2_PO_4_	85.1	0.0	0.0
KNO_3_	949.8	1013.0	1013.0
MgSO_4_·7H_2_O	184.9	0.0	0.0
NH_4_NO_3_	824.84	780	780
NH_4_MgPO_4_·6H_2_O (struvite)	_	_	153.4

All media were adjusted to pH 5.8 with 1 M KOH. The media were sterilized using an autoclave before use (120 °C, 30 min). From the different media, 20 mL was added into 5 cm deep well Petri dishes (Thermo Fisher). Seeds of *A. thaliana* (Col-0) were surface-sterilized with 20% dilution of 3.6% sodium hypochlorite (ParoZone, Henkel) for 20 min and washed six times with sterile double-distilled water, and a single seed was placed in the middle of each Petri dish on the surface of the medium. The Petri dishes were sealed with a Micropore surgical tape (3M) to allow for gas exchange.

The sown seeds were incubated at 4 °C overnight to improve and synchronize germination.^50^ Plants were grown on a 21 °C-16 h day and 20 °C-8 h night cycle in an MLR-352-PE growth chamber (Panasonic) for 21 days. Petri dishes with plants were imaged by using an iPhone 11 (Apple) camera at a fixed distance of 12 cm from the bench. Using a custom Python script, the leaf surface area was extracted using the OpenCV^51^ function inRange. The resulting mask was quantified in number of pixels to produce an estimate of the leaf surface area.^52^ The normality of the distribution of the data was visually assessed via a Q–Q plot and statistically via a Shapiro–Wilk test (*p* = 0.19). The *p* value, although low, does not fall below the significancy threshold of *p* < 0.05. However, together with the visual verification of the Q–Q plot, we rejected the null hypothesis that the data was normally distributed. To assess whether the samples came from the same distribution, a Kruskal–Wallis one-way analysis of variance was performed followed by a Dunn’s test. A standard threshold in biology of *p* < 0.05 was used to reject the null hypothesis that samples originated from the same distribution.

## Results and Discussion

This work proposes a three-step process for the recovery of phosphate from undiluted wastewaters (i.e., at the production point). In the first step, the Mg–Fe layered double hydroxide (LDH) is used as the adsorbent of phosphate. In the second step, phosphate is released back into the enriched solution through desorption. In the last step, struvite is precipitated to be used as a fertilizer. To prove the process, the mechanism, kinetics, and thermodynamics of adsorption of phosphate on Mg–Fe LDH is presented herein, followed by the kinetics of the desorption process. Finally, we demonstrate the efficient recycle of the recovered phosphate in the form of struvite as a fertilizer.

### Step 1: Adsorption of Phosphate

The separation of phosphate species from aqueous solution with a phosphate concentration of 500 mg/L (similar to human urine) was studied using Mg–Fe layered double hydroxide (LDH) as an adsorbent. This material was selected due to its high positive ζ-potential between pH 4 and 10 and its fast adsorption kinetics toward phosphate uptake.^34,41,,54^ LDH materials are composed of two-dimensional lamellar mixed hydroxides, represented by the general formula [M_1–*x*_^+2^M_x_^+3^(OH)_2_]^*x*+^A_*x*/*m*_^*m*–^·*n*H_2_O, where M^+2^ and M^+3^ are divalent and trivalent cations, respectively. A^*m*–^ represents the incorporated anions in the interlayer space, and the value of *x* is equal to the molar ratio of M^+3^/(M^+2^ + M^+3^). For the Mg–Fe LDH material, the general formula is [Mg_0.75_Fe_0.25_(OH)_2_]^0.25+^(CO_3_)_0.125_^2–^·*n*H_2_O, where the interlayer ion is CO_3_^2–^. Other metal compositions such as Mg–Al, Zr–Al, and Zn–Fe were also explored, but they presented a lower phosphate capacity in the first cycle.

The normal pH range of fresh urine is 4.5–7.5,^55^ with average values around pH ≈ 6.^56,57^ At a pH of 6, the adsorption capacity of Mg–Fe LDH using an initial phosphorus concentration of 500 mg/L is 78.6 mg/g. Previous work using LDH materials for phosphate uptake reported adsorption capacity values of 13 mg/g for Mg–Fe LDH^54^ (interlayer ion: NO_3_^–^; experimental conditions: *C*_0_: 16.3 mg/L, 1 g/L, and pH: 7.4) and 21.1 mg/g for Mg–Fe LDH^43^ (interlayer ion: Cl^–^; experimental conditions: *C*_0_: 20 mg/L, 0.3 g/L, and pH: 5.5). However, direct comparison of adsorption capacities cannot be carried out due to the lower concentration range of phosphorus (<20 mg/L) used in the literature, which are typical values of inlet wastewater streams.^41−44^ Other materials, such as magnesium amorphous calcium carbonate, were evaluated at a higher phosphorus concentration, and an adsorption capacity value of 62 mg/g^38^ was obtained (experimental conditions: *C*_0_: 465 mg/L, 5 g/L, and pH: 10).

**Figure 2:**
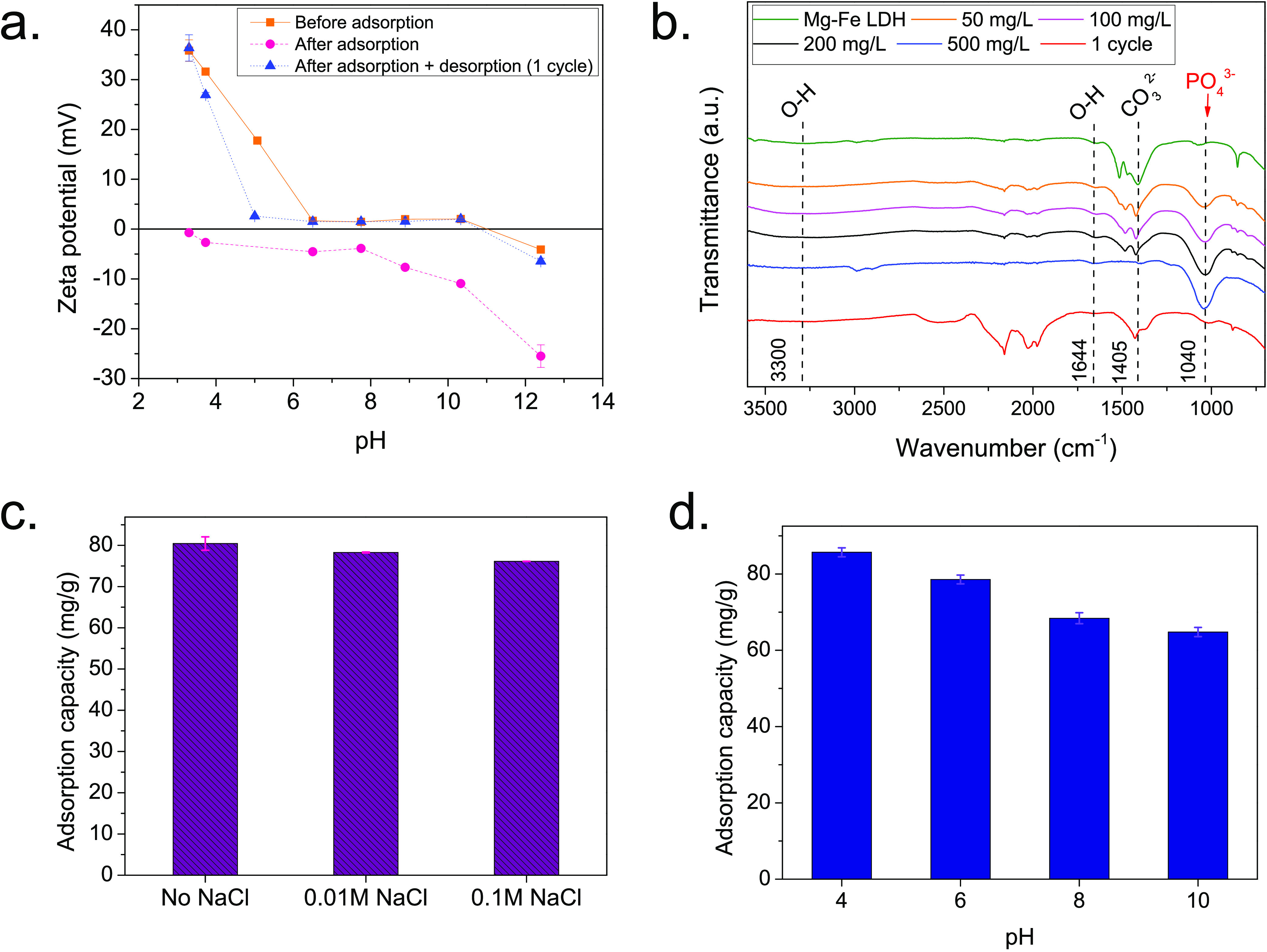
(a) Mg–Fe LDH ζ-potential analysis before and after phosphate adsorption and after 1 cycle (adsorption + desorption); (b) FTIR spectra of fresh Mg–Fe LDH, after adsorption with different phosphate initial concentrations (*C*_0_ = 50, 100, 200, 500 mg/L) and after 1 cycle (*C*_0_ = 500 mg/L); (c) the effect of ionic strength on phosphate adsorption onto Mg–Fe LDH at pH = 4.8; and (d) the effect of pH on phosphate adsorption onto Mg–Fe LDH. Adsorbent dosage = 5 g/L, *C*_0_ = 500 mg/L, *T* = 25 °C, *t* = 1 h, and pH = 4.8.

**Figure 3:**
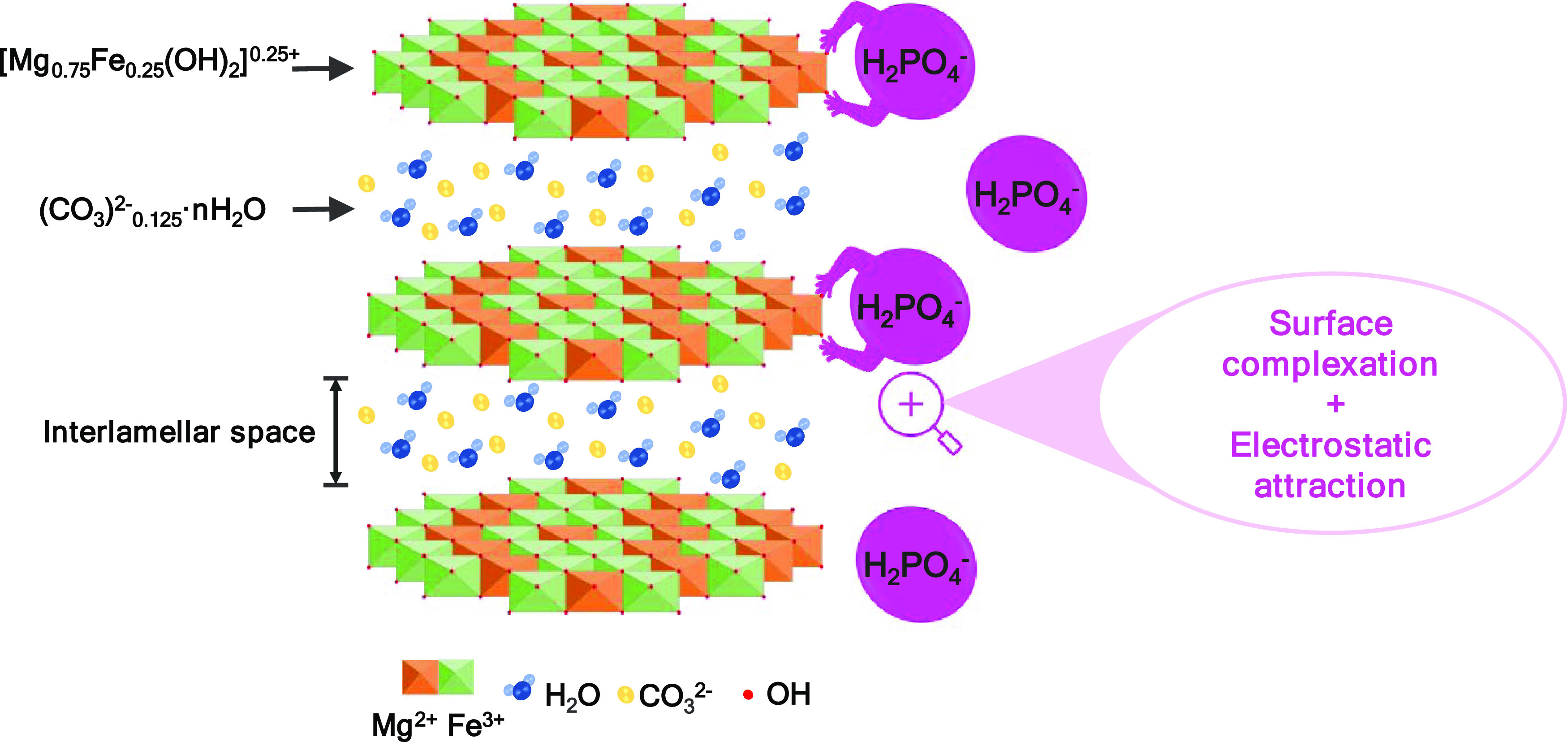
Schematic illustration of the layered double hydroxide material structure and the phosphate adsorption mechanism onto the Mg–Fe LDH adsorbent.

At the pH range of fresh urine, the surface of Mg–Fe LDH is positively charged according to the zeta-potential analysis of the fresh material (shown in Figure 2a) suggesting a potential electrostatic attraction of the phosphate species (mainly H_2_PO_4_^–^ at pH < 7), negatively charged under these conditions as depicted in Figure 3. In fact, the phosphate solution used for the adsorption experiments presents a ζ-potential value of −28.6 mV at a pH value of 4.8. ζ-Potential measurements after adsorption confirm such electrostatic attraction as the surface of the Mg–Fe LDH material is neutralized (i.e., slightly negative ζ-potential values at acidic pH). The high isoelectric point close to pH ≈ 10.5 of the Mg–Fe LDH suggests that this separation will occur under varying urine conditions (and compositions). In addition to electrostatic interactions, phosphate species also chemisorb onto the magnesium and iron ions at the surface of LDH by replacing the hydroxyl ions, forming a surface complex M–O–P as illustrated in Figure 3 and evidenced by FTIR spectroscopy. Figure 2b shows the FTIR spectra before and after phosphate adsorption. The Mg–Fe LDH material presents bands at 3300 cm^–1^ which represents O–H stretching vibrations from structural hydroxyl groups, at 1644 cm^–1^ which indicates O–H bending vibration from interstitial water molecules, and at 1405 cm^–1^ which shows the presence of CO_3_^2–^ in the interlayer region of the material.^58−61^ A new peak appears after phosphate adsorption onto Mg–Fe LDH at 1040 cm^–1^, assigned to the symmetrical stretching vibration of PO_4_.^3−54,62^ The intensity of this phosphate band increases with an increase in the initial phosphate concentration. The peaks shown between 2350 and 2000 cm^–1^ are a combination of atmospheric CO_2_ and moisture from air. The CO_2_ asymmetric stretching mode is assigned between 2200 and 2350 cm^–1^,^63−65^ and the water band is located between 2000 and 2120 cm^–1^, which is due to the coupling of O–H–O scissors bending and a broad liberation band in the near-infrared.^66,67^

Increasing the electrolyte concentration by adding different concentrations of NaCl has a very small effect on the phosphate adsorption capacity of the Mg–Fe LDH (Figure 2c). This insensitivity to ionic strength is expected when the adsorption mechanism is via chemisorption through the formation of surface complexes as shown above. However, the small decrease in adsorption capacity observed when increasing the ionic strength can be associated with the competitive electrostatic adsorption of the electrolyte and the phosphate. The combination of both phosphate adsorption mechanisms, electrostatic interaction and chemisorption, has been reported earlier in similar materials such as MgAl-CO_3_ and MgFe-Cl^–^ LDHs,^54^ including the ion exchange between phosphate and the chloride interlayer species as evidenced by an increase of the interlayer space by X-ray diffraction (XRD).^43^ In this case, XRD (Figure S2) shows a complete loss of crystallinity during the adsorption process.

Similar conclusions are obtained by studying the effect of pH on the phosphate adsorption capacity (Figure 2d). The highest phosphate adsorption capacity is achieved at the lowest pH value of 4, as the Mg–Fe LDH surface is positively charged (see Figure 2a), favoring the electrostatic attraction between the negatively charged H_2_PO_4_^–^ and positively charged LDH surface. Increasing the pH value has a detrimental effect on the phosphate adsorption capacity due to two parallel effects. At pH values of 6 and above, the LDH surface is negatively charged, reducing its electrostatic interaction with the negatively charged phosphate anions. In addition, the increase in concentration of hydroxyl groups at high pH leads to a competing adsorption with the phosphate ions for the Mg–Fe LDH adsorption sites, also decreasing the phosphate adsorption capacity.

**Figure 4:**
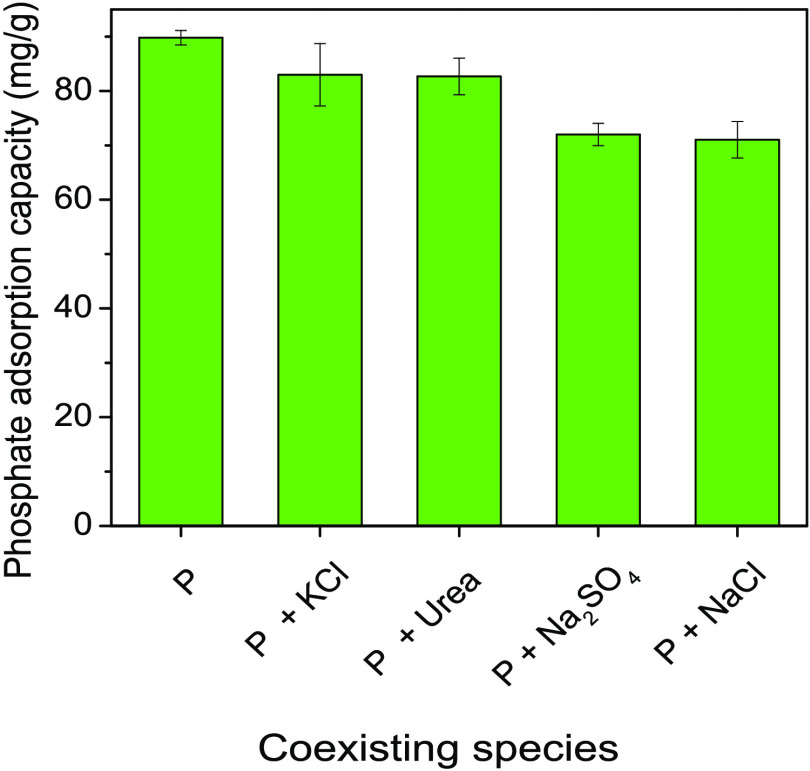
Effect of coexisting species commonly present in urine on the phosphate adsorption capacity of Mg–Fe LDH. Adsorbent dosage = 5 g/L, *T* = 25 °C, *t* = 1 h, *C*_0_ (PO_4_–P) = 900 mg/L, *C*_0_ (KCl) = 1600 mg/L, *C*_0_ (urea) = 25,000 mg/L, *C*_0_ (Na_2_SO_4_) = 2300 mg/L, and *C*_0_ (NaCl) = 4600 mg/L.

It is important to note that when using real urine waste streams, other components present in the complex mixture might adsorb competitively with phosphate species. In order to evaluate the effect of coexisting species on the phosphate adsorption, adsorption experiments were performed by adding additional compounds commonly present in urine.^45^ The study focused on those components with the highest concentration in urine, as it is not intended to be an exhaustive study. Thus, KCl (1600 mg/L), urea (25,000 mg/L), Na_2_SO_4_ (2300 mg/L), and NaCl (4600 mg/L) were added to the model phosphate aqueous solutions. As can be seen in Figure 4, the addition of KCl and urea species to the aqueous solutions does not significantly affect the adsorption capacity of Mg–Fe LDH toward phosphate. However, the presence of anionic species such as SO_4_^2–^ and Cl^–^ has a detrimental effect, decreasing the phosphate adsorption capacity by ∼18%. This is due to the fact that SO_4_^2–^ and Cl^–^ are negatively charged species that compete together with phosphate for the adsorption sites onto the Mg–Fe LDH surface in agreement with previous studies.^41,43^

**Figure 5:**
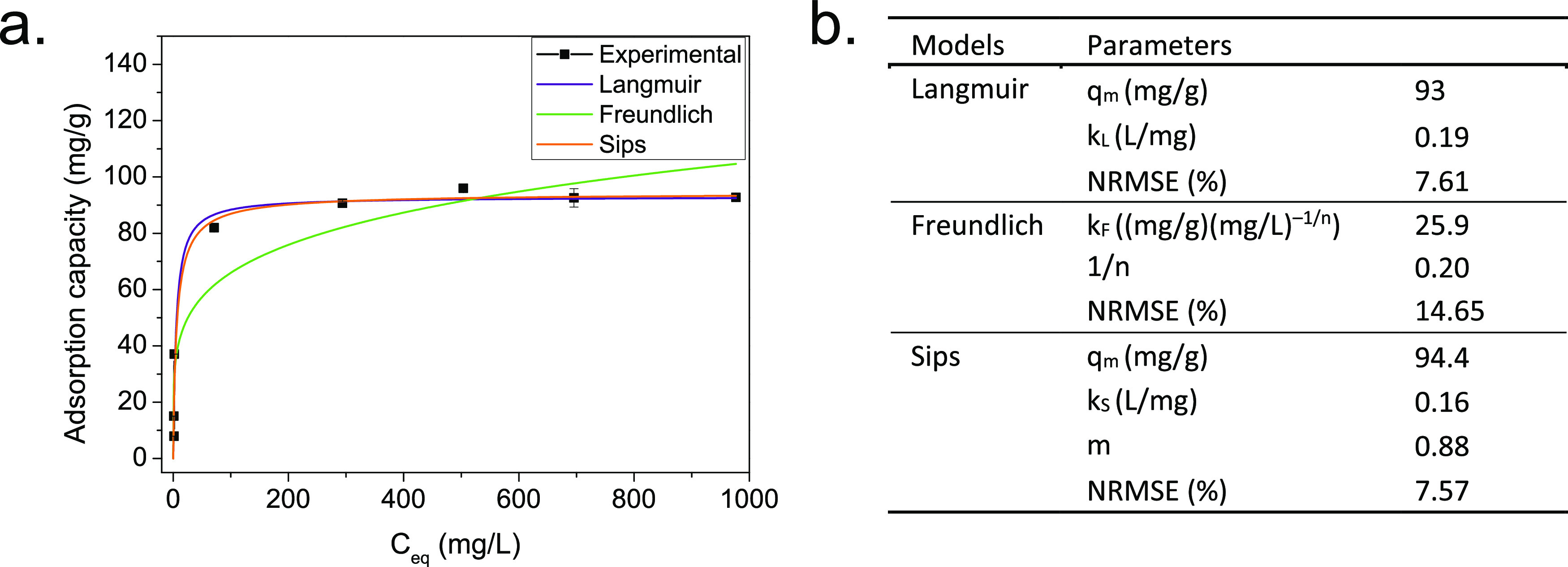
(a) Adsorption isotherm for Mg–Fe LDH, *C*_0_ = 40–1500 mg/L, adsorbent dosage = 5 g/L, *t* = 1 h, *T* = 25 °C, and pH = 4.8. (b) Parameters and coefficients of Langmuir, Freundlich, and Sips isotherm models of phosphate adsorption onto Mg–Fe LDH.

By varying the initial concentrations of phosphate between 40 and 1500 mg/L and keeping constant the amount of Mg–Fe LDH, the isotherms of adsorption at 25 °C were obtained as shown in Figure 5. The highest adsorption capacity obtained in this material is ∼90 mg/g. Increasing the initial concentration did not further increase this value, suggesting that the material is saturated. It is important to note that saturation is achieved with initial phosphorus concentrations similar to the ones found in undiluted urine (500 mg/L) being possible to exploit the full capacity of the Mg–Fe LDH.

The experimental data were fitted to the Langmuir (eq 4), Freundlich (eq 5), and Sips (eq 6) adsorption models.
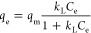
4
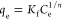
5
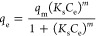
6where *q*_m_ is the maximum adsorption capacity (mg/g) and *K*_L_ is the Langmuir constant (L/mg); *K*_F_ is the Freundlich constant ((mg/g)(mg/L)^−1/*n*^) and 1/*n* is the heterogeneity factor; *K*_s_ is the Sips constant (L/mg) and *m* is the exponent of the Sips model that can be used to describe the heterogeneous system.

Since the experimental data are fitted through nonlinear regression, the normalized root-mean-squared error (NRMSE %) is used for comparison, as shown in eq 7.
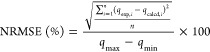
7where *q*_calcd,*i*_ is each value of *q* predicted by the fitted model; *q*_exp,*i*_ is each value of *q* measured experimentally; *n* is the number of experimental points; and *q*_max_ and *q*_min_ are the maximum and minimum values of the measured data, respectively.

Langmuir and Sips models fit the data well with similar NRMSE values (7.61 and 7.57%, respectively), confirming a monolayer adsorption as expected for chemisorption. Although the Langmuir isotherm assumes a uniform surface with finite identical sites and no interaction between adjacent molecules on neighboring sites^68^ and the Sips isotherm is used in heterogeneous systems, the fact that the “*m*” constant in the latter model (0.88) approaches 1 reduces the Sips model to the Langmuir isotherm.^68^ Indeed, both models predict similar adsorption capacities (*q*_m_) comparable to the experimental value. On the other hand, as expected, the Freundlich model, not restricted to monolayer adsorption and assuming a heterogeneous surface, with nonuniform distribution of the adsorption sites energies,^68^ does not fit the data well. In addition, the low “1/*n*” constant value of 0.2 in the Freundlich model suggests monolayer adsorption.

**Figure 6:**
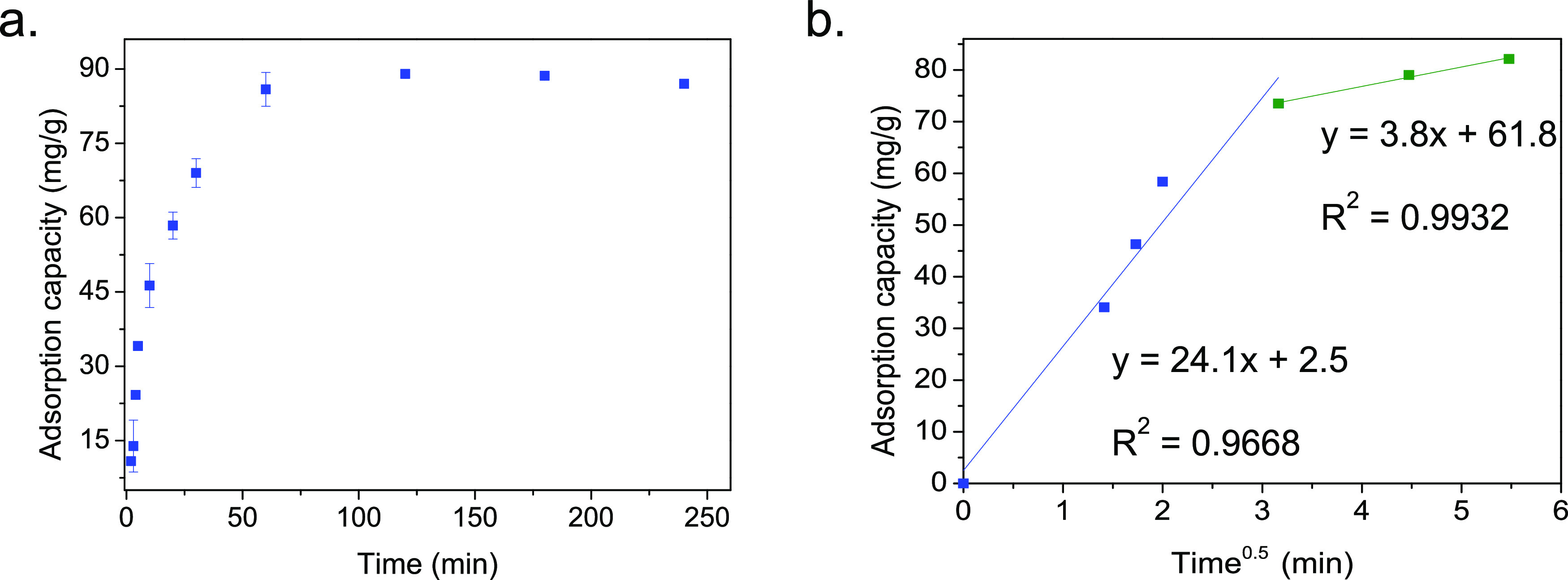
(a) Kinetic study of the adsorption capacity as a function of time of phosphate onto Mg–Fe LDH, and (b) kinetic data fitting to the Weber and Morris model. Phosphate initial concentration: 500 mg/L, adsorbent: 5 g/L; *t*: 5 h; pH: 4.8; *T*: 25 °C.

The rate of adsorption was evaluated by using an initial phosphate concentration of 500 ppm (Figure 6a). As expected, the adsorption rate is at its maximum value during the first 30 min, after which it decreases slightly, reaching equilibrium at an adsorption capacity of 85 mg/g within 1 h. To evaluate the rate limiting step in the process, the kinetic data was fitted to the Weber and Morris model^69^ (Figure 6b), which assumes internal mass transfer limitations where film diffusion is not significant or only significant for a very small period at the beginning of diffusion. The model is described in eq 8.
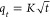
8where *K* (mg/g·min^0.5^) is the intraparticle diffusion rate; which is obtained from the slope in the plot of *q_t_* versus *t*^0.5^; and *q*_*t*_ (mg/g) is the adsorption capacity at time *t* (min).

The multilinearity of the fitting indicates that internal pore diffusion limits the adsorption rate.^70−73^ The higher intraparticle diffusion rate value in the first linear region of the graph represents the internal diffusion into pores of bigger width values (*K*_1_ = 24.1 mg/g·min^0.5^ > *K*_2_ = 3.8 mg/g·min^0.5^). Then, the internal diffusion of phosphate molecules continues into smaller pore sizes, which results in a decrease in the “*K*” parameter in the second linear region.

### Step 2: Phosphate Desorption and Adsorbent Reusability

**Figure 7:**
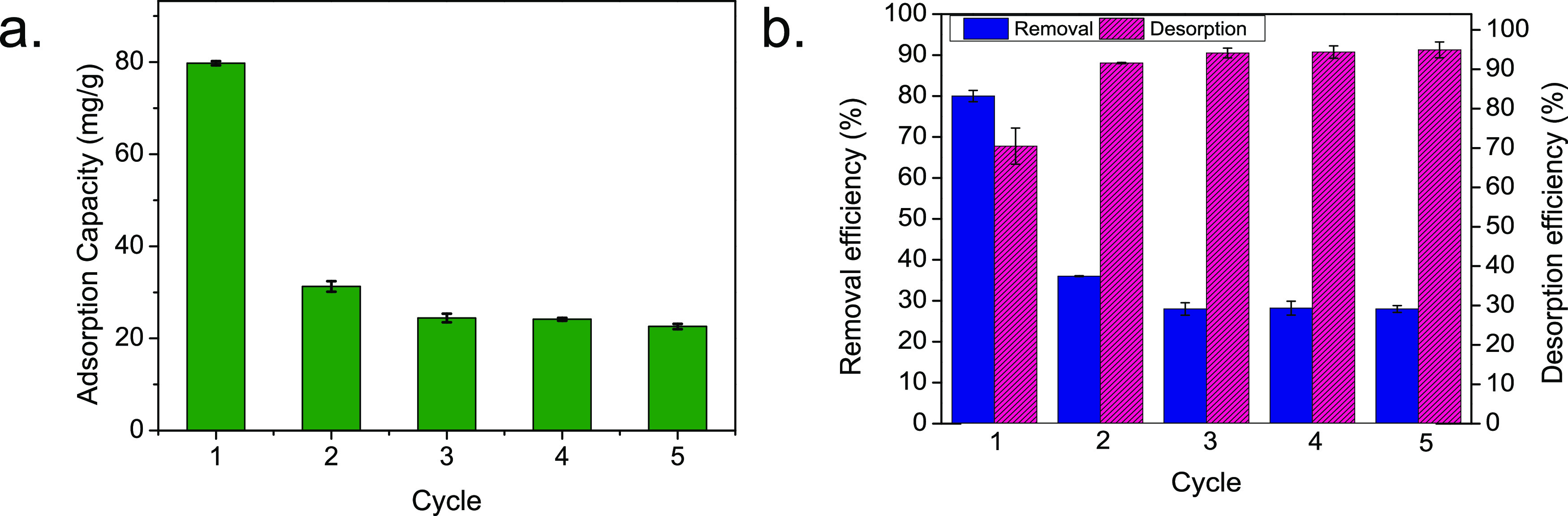
(a) Adsorbent reusability throughout 5 adsorption–desorption cycles. (b) Recovery and desorption efficiency for 5 adsorption/desorption cycles. Adsorption: adsorbent dosage: 5 g/L; *C*_0_: 500 mg/L; *T*: 25 °C, *t*: 1 h, pH: 4.8. Desorption: *t*: 30 min, 0.5 M NaOH, *T*: 25 °C.

After adsorption, the Mg–Fe LDH adsorbent is regenerated by releasing phosphate. For this, the phosphate-loaded adsorbent was first separated by using centrifugation. Phosphate desorption was carried out in batch by redispersing the adsorbent loaded with phosphate into 10 mL of a 0.5 M NaOH solution. The presence of concentrated NaOH displaces phosphate from the adsorption sites. Then, the supernatant is collected to determine the phosphate release back into the solution. The same desorption solution was used in the 5 desorption cycles in order to increase the concentration of phosphate, since a concentration of 50 mg/L PO_4_–P has been reported as the threshold for the feasibility of struvite precipitation.^74^ In the first cycle, 401 ppm of the original 500 ppm phosphate solution was removed by the Mg–Fe LDH (80% removal efficiency at an adsorbent concentration of 5 g/L), releasing 295 ppm back into the alkaline solution (71% desorption efficiency). The recovery efficiency dramatically drops to values ∼30% in consecutive cycles due to the lower adsorption capacity (Figure 7).

Consecutive adsorption/desorption cycles show a decrease in the adsorption capacity of the Mg–Fe LDH after the first cycle although the desorption efficiency is maintained at ∼90% (Figure 6b). The drop in adsorption capacity is partially associated with the lack of full desorption as confirmed by the low phosphate intensity peak present in the FTIR spectra after the first cycle (adsorption and desorption) as shown in Figure 2b.

However, the main loss in adsorption capacity after the first cycle is related to the loss of the Mg–Fe LDH crystallinity due to hydration in aqueous solutions, leading to a stacking disorder in the layered structure, which is translated into a decrease of the ζ-potential from the original 17.8 to 2.7 mV at pH = 4.8, negating the electrostatic interaction of phosphate in consecutive adsorption cycles. This is confirmed by performing a blank initial experiment in water only (i.e., suspension of the fresh Mg–Fe LDH in water) and then utilizing the material for phosphate adsorption. After exposing the material to water, the initial adsorption capacity decreases from 85 to 35 mg/g in the first adsorption cycle. Similarly, the amorphous XRD pattern after adsorption (Figure S2) also shows the loss of crystallinity of the Mg–Fe LDH material. It is important to highlight that after this initial decrease of adsorption capacity in the first cycle, the material remains stable in consecutive cycles with high desorption efficiency.

Despite the loss of the crystal structure, leaching of Mg or Fe does not take place either during the hydration of the material or during the adsorption–desorption cycles, as shown by inductively coupled plasma-optical emission spectrometry (ICP-OES) analyses. Table 2 shows the amount of Mg^2+^ and Fe^3+^ ions in the supernatant samples after the blank experiment and the five adsorption cycles expressed as percentage of LDH dissolved in solution (wt %).

**Table 2:** ICP-OES Analysis of the Supernatant Samples after the Blank Experiment and Adsorption Cycles

adsorption cycle	Mg^2+^ (wt % of LDH dissolved)	Fe^3+^ (wt % of LDH dissolved)	desorption cycle	Mg^2+^ (wt % of LDH dissolved)	Fe^3+^ (wt % of LDH dissolved)
blank water	1.1	0	1st	0.0002	0.0053
1st	0.4	0	2nd	0.0001	0.0078
2nd	0.05	0	3rd	0.0001	0.0048
3rd	0.02	0	_	_	_
4th	0.02	0	_	_	_
5th	0.008	0	_	_	_

### Step 3: Precipitation of Struvite and Its Use as a Fertilizer

The phosphate-enriched solution after 5 consecutive cycles, with a concentration of 842 mg/L of P, was used for the precipitation of phosphorus in the form of struvite (MgNH_4_PO_4_·6H_2_O), commonly used in agriculture as a slow-release fertilizer. For this, magnesium and ammonium salts (MgCl_2_ and NH_4_Cl) were added to the recovered phosphate solution at a molar ratio of Mg/NH_4_/PO_4_ = 1:1:1 and at a pH = 8.5 adjusted with 0.1 M HCl. After 30 min, 98.7% of phosphate is precipitated according to eq 9.
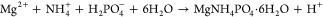
9

**Figure 8:**
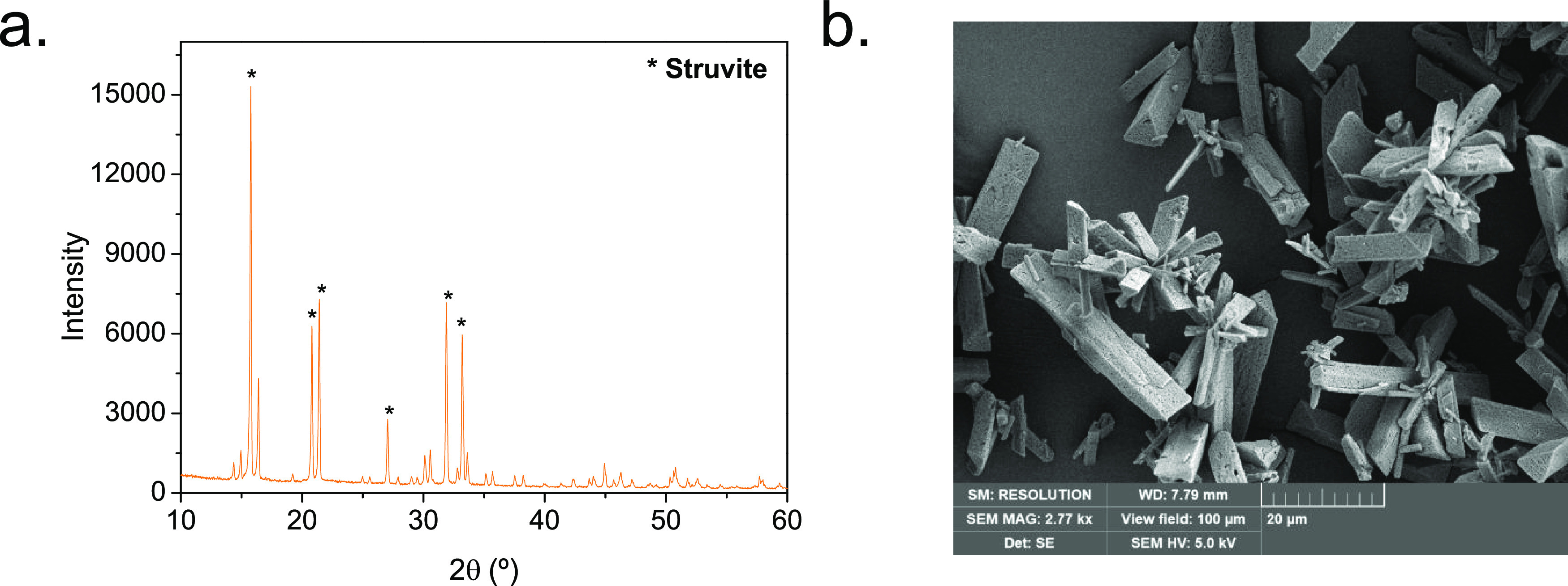
Characterization of struvite obtained from the precipitation process: (a) XRD pattern and (b) SEM image. Struvite precipitation experimental conditions: pH = 8.5, Mg/NH_4_/PO_4_ molar ratio of 1:1:1, *t* = 30 min.

The sustainability of the process can be further enhanced by using naturally abundant magnesium sources, such as MgO and MgCO_3_. The obtained crystals correspond to pure struvite as confirmed by its XRD pattern (Figure 8a) that matches with the standard for this material from the diffraction database of the International Centre for Diffraction Data of struvite (PDF# 71-2089) as well as the rodlike irregular crystals with aggregates at the edges observed by SEM (Figure 8b). No evidence of low-soluble magnesium-based salts such as MgO or MgCO_3_ was observed,^75^ probably related to the high initial concentration of phosphate during the crystallization process.

Recovered struvite can be used as a sustainable alternative to conventional phosphate rock-based fertilizers for plant growth.^76^ To assess its feasibility, a plant growth assay was carried out using a commonly used model organism to study plants, *A. thaliana*. This plant can be grown in axenic culture on a half strength Murashige and Skoog (1/2 MS)^77^ (Table 1), a nutrient mixture containing phosphates, and other important plant nutrients. In this experiment, the effect on plant growth of three conditions is compared: (1) Half strength MS, containing all required nutrients and is used as a positive control for plant growth; (2) Mg- and P-deficient medium (MgP-DM), a nutrient medium based on half strength MS, lacking core macronutrients including magnesium and phosphorus, and this is used as a negative control for plant growth; and (3) MgP-DM + Struvite (MgP-DMS). The MgP-DM was calculated so that the addition of struvite almost equalizes the ion composition of the nutrient mix to half strength MS.

**Figure 9:**
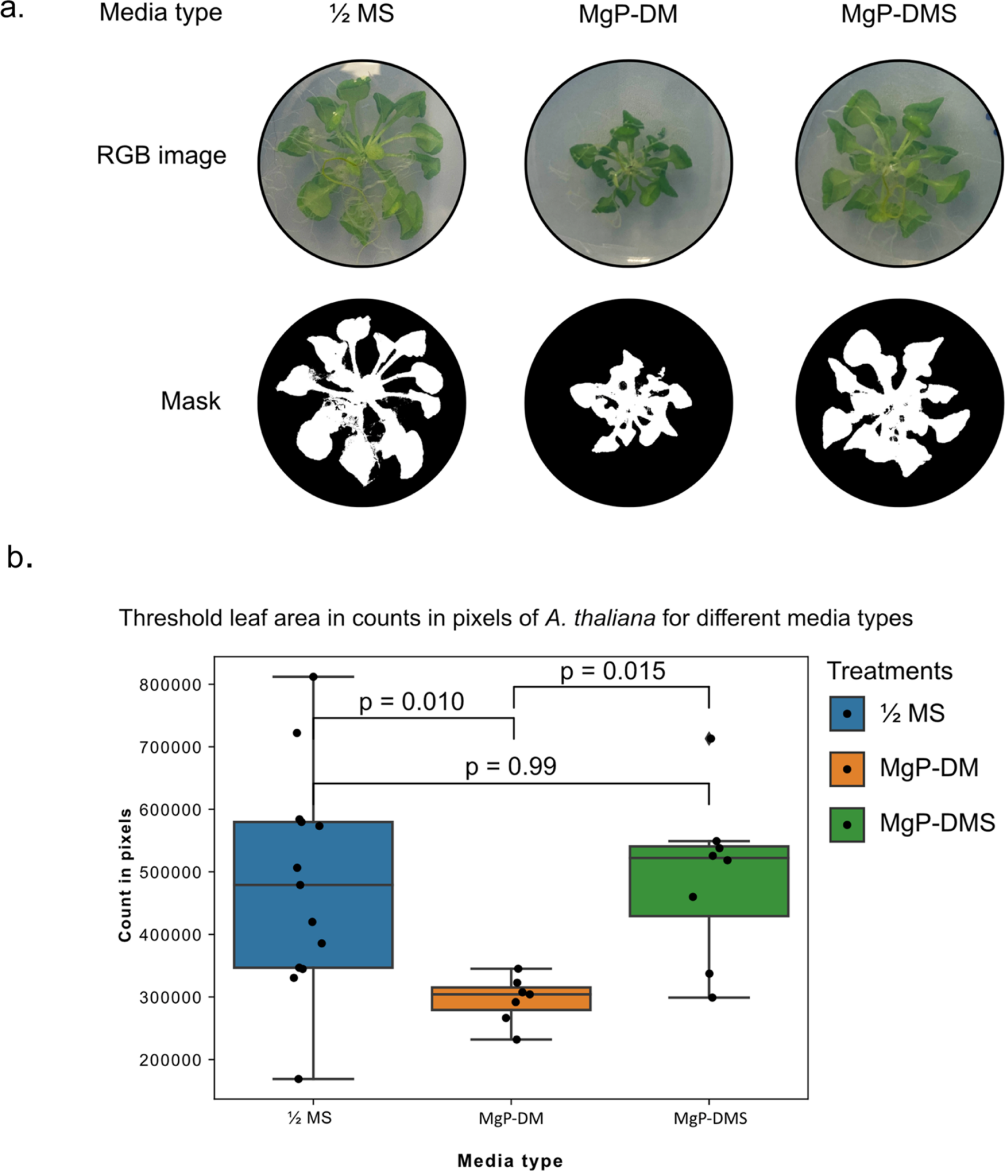
(a) Color thresholding was used to determine the leaf surface area of *A. thaliana* from images (representatives shown: RGB images (23% brightness, + 64% contrast for clarity) and threshold masks shown); (b) the number of white pixels was then counted from the overlay and used to estimate the leaf surface area. The leaf surface area was compared between the following conditions: 1/2 MS (a standard growth medium for *A. thaliana*), MgP-DM (a reduction of nutrients), and MgP-DMS (similar to commercial 1/2 MS). The distribution of the data is shown in the boxplot in section B (*n* = 7). *P* values are shown for the Dunn test for differences between conditions.

Plants were grown post sowing for 21 days under 21 °C-16 h day and 20 °C-8 h night conditions. Images of the plant leaves were captured at a fixed distance. The leaf surface area was measured from the images using a color-based thresholding method (Figure 9a) and quantified in number of pixels (Figure 9b). The probability (*p* value) that these distributions are not different was calculated using a Kruskal–Wallis^78^ test and the post hoc Dunn test.^79^ In plant growth assays, a *p* value of <0.05 is generally considered a significant difference. The Kruskal–Wallis test reports a *p* value of 0.007, indicating that the mean ranks of at least one group are different to the other conditions. To determine which groups are different, a pairwise comparison was performed using a Dunn test. Using the significance threshold of *p* < 0.05, half strength MS was not different to MgP-DMS, MgP-DM was different to MgP-DMS, and MgP-DM is different to half strength MS (Figure 9b). These results show that the addition of struvite to MgP-DM is enough to recover the plant growth phenotype achieved with 1/2 MS growth media. Importantly, this *in vitro* test system uses much less overall additive per plant (0.003 g compared to 2.86 g per plant in soil^80^), making it suitable for testing larger numbers of plants for a given quantity of additive.

## Conclusions

This work demonstrated the efficient recovery and reusability of phosphate as a fertilizer from aqueous solutions with concentrations similar to those found in human urine using Mg–Fe layered double hydroxide (LDH) material. The initial phosphate uptake mechanism is a combination of electrostatic attraction and surface complexation as confirmed by the high isoelectric point of the adsorbent (pH ≈ 10.5) through ζ-potential analysis and the presence of PO_4_^3–^ band peaks in the FTIR spectra. After the first cycle, there was a decrease in the adsorption capacity of the Mg–Fe LDH material caused by the lack of full desorption of phosphate from its surface and the stacking disordering of the layered structure in the presence of water. The reusability of the material is demonstrated in its use in five consecutive adsorption/desorption cycles, where the removal and desorption efficiency are kept at approximately 30 and 90%, respectively. Through consecutive adsorption and desorption cycles, the phosphate solution is enriched, making possible its chemical precipitation as struvite by minimizing the amount of used chemicals. Finally, we demonstrated the use of the recovered struvite for healthy plant growth, successfully replacing rock-originated phosphate in growth media effectively, mimicking the waste-to-nutrient circular cycle in nature.

## Supporting Information Available

UV–vis calibration curve for phosphate concentration determination (Figure S1) and XRD patterns of Mg–Fe LDH before and after adsorption (Figure S2) (PDF)
