# Supplementary material for: Phosphate Recovery from Urine-Equivalent Solutions for Fertilizer Production for Plant Growth
Source: ACS Sustain Chem Eng. 2023 Nov 1;11(45):16074–86. doi: 10.1021/acssuschemeng.3c03146 (PMC10647925; doi:10.1021/acssuschemeng.3c03146)
Supplement: Supplementary file 1 — sc3c03146_si_001.pdf [file sc3c03146_si_001.pdf]

Supporting information

Phosphate recovery from urine-equivalent solutions for fertiliser production for plant growth

*Marina Avena Maia[1], Olaf Prosper Kranse[2], Sebastian Eves-van den Akker[2] and Laura Torrente-Murciano[1]**

[1] Department of Chemical Engineering and Biotechnology, University of Cambridge, Philippa Fawcett Drive, CB3 0AS, Cambridge, UK

[2]Crop Science Centre, Department of Plant Sciences, University of Cambridge, CB3 0LE, Cambridge UK

This SI contains 2 pages and 2 figures

Figure S1: UV-vis calibration curve using a colorimetric method for phosphate concentration determination in aqueous solutions...2

Figure S2: XRD patterns of Mg-Fe LDH before and after adsorption..2

Vanadomolybdophosphoric acid colorimetric method for the determination of phosphorus in aqueous solutions

A colorimetric method was developed based on the APHA Standard Colorimetric Method 4500-P C to determine the concentration of phosphate (PO_4-P) in aqueous solutions using UV-vis spectroscopy. In a phosphate-containing solution, ammonium molybdate reacts under acidic conditions to form a heteropoly acid, molybdophosphoric acid. In the presence of vanadium, yellow vanadomolybdophosphoric acid is formed. The absorbance intensity at 421 nm is proportional to phosphorus concentration in agreement with the Lambert Beer's law. For the calibration curve (Figure S1), solutions with a phosphate concentration between 1 and 50 mg/L were used.

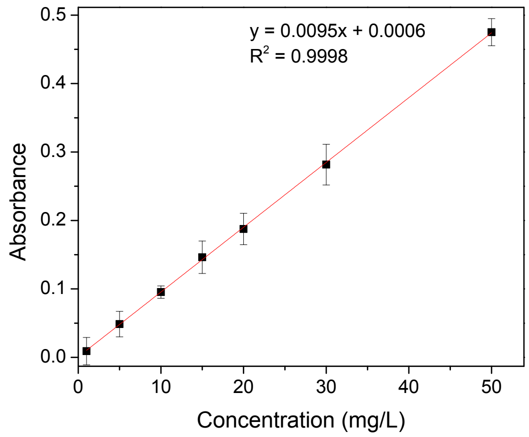

Figure S1: UV-vis calibration curve using a colorimetric method for phosphate concentration determination in aqueous solutions.

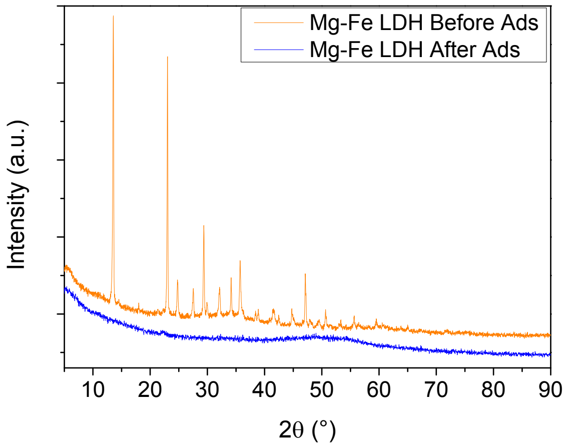

Figure S2: XRD patterns of Mg-Fe LDH before and after adsorption.
